# Longitudinal Study of Antibiotic Resistance of Staphylococci from Cases of Subclinical Mastitis in Sheep in Greece: Incidence and Risk Factors

**DOI:** 10.3390/antibiotics12121703

**Published:** 2023-12-07

**Authors:** Charalambia K. Michael, Daphne T. Lianou, Katerina Tsilipounidaki, Zoe Florou, Natalia G. C. Vasileiou, Vasia S. Mavrogianni, Efthymia Petinaki, George C. Fthenakis

**Affiliations:** 1 Veterinary Faculty, University of Thessaly, 43100 Karditsa, Greece; 2 University Hospital of Larissa, 41110 Larissa, Greece; 3 Faculty of Animal Science, University of Thessaly, 41110 Larissa, Greece

**Keywords:** antibiotic resistance, biofilm, goat, mastitis, methicillin, risk factor, sheep, *staphylococcus*

## Abstract

The present paper extends a previous publication on a field study of subclinical mastitis in sheep and focuses on the following laboratory characteristics of the staphylococcal isolates: antibiotic resistance and association with biofilm formation. The specific objectives of the present study were (a) to describe the incidence of isolation of antibiotic-resistant staphylococci from cases of mastitis throughout the milking period in dairy sheep flocks and (b) to identify relevant risk factors, which would contribute to the sustainable control of the infection. Staphylococcal isolates from subclinical mastitis were evaluated for antibiotic resistance to 18 antibiotics. Antibiotic resistance was detected in 57 of the 179 staphylococcal isolates from subclinical mastitis (31.8%). Resistance was recorded against 11 antibiotics, most often against ampicillin (63.2% of resistant isolates), penicillin (63.2%) and tetracycline (47.4%). Isolates resistant to ampicillin and penicillin were recovered in all 12 farms. Twenty-one multidrug-resistant isolates (11.7%) were also recovered. The incidence risk of isolation of staphylococci resistant to at least one (any) antibiotic throughout the study period was 23.8%. The incidence risk of isolation of staphylococci resistant to oxacillin was 5.0%; that of isolation of multidrug-resistant staphylococci was 8.8%. With regard to increased incidence risk of isolation of staphylococci resistant to at least one (any) antibiotic and increased incidence risk of isolation of staphylococci resistant to oxacillin, the omission of anti-staphylococcal mastitis vaccination of ewes emerged as a risk factor. With regard to increased incidence risk of isolation of multidrug-resistant staphylococci, the following variables emerged as risk factors: (a) higher number of antibiotics used on the farm for the treatment of mastitis and (b) younger age of lambs taken away from their dam. Most biofilm-forming antibiotic-resistant staphylococci were recovered from farms where anti-staphylococcal mastitis vaccination was not applied (55.9% versus 44.1% from farms where anti-staphylococcal mastitis vaccination was applied).

## 1. Introduction

Staphylococci are the most frequent causal agents of ovine mastitis, which is responsible for significant adverse financial and welfare effects [1]. A recent extensive study carried out in Greece revealed that sheep farmers considered mastitis as the most important health problem on their farms [2]. Effective treatment of the infection is important in order to limit the infection and potentially to restore the health of the affected animals. Hence, there is merit in studying and monitoring the antibiotic resistance patterns of these bacteria, in order to provide updated information regarding effective control of the infection.

Studies performed in Europe and neighbouring countries into the antibiotic resistance patterns of staphylococci associated with mastitis in sheep have not indicated, in general, problems of reduced susceptibility to antibiotics. Most of the relevant studies have reported resistance rates up to 45%, which depended on the staphylococcal species (lower resistance rate among *S. aureus*, higher among coagulase-negative isolates) and the antibiotic concerned (higher resistance rate to penicillins) [3,4,5,6,7,8,9,10]. However, differentiations from the above general findings have also been reported: for example, in a study by Azara et al. [11], a higher resistance rate of *S. aureus* (rather than coagulase-negative isolates) against tetracycline (rather than penicillins) was reported, whilst Regecová et al. [12] reported particularly high rates of resistance of *S. chromogenes* to penicillins and tetracycline.

The literature has provided information regarding the antibiotic resistance patterns of causal pathogens isolated as part of examination of clinical cases of mastitis or of cross-sectional studies on mastitis prevalence and aetiology. A topical search in the Web of Science using the search string [*resistance* AND (antibiotic* OR antimicrobial*) AND (*sheep* OR ewe*) AND *mastitis* AND *incidence*] returned seven documents, a detailed examination of which revealed that none included a longitudinal study of antibiotic resistance on the study farms. A similar search using the search term (*cattle* OR cow*) in lieu of (*sheep* OR ewe*) in the above string returned 88 documents; among these, relevant longitudinal studies, performed in the past or more recently, were identified (e.g., [13,14,15]).

The present work refers to a longitudinal study that had been carried out in Greece from November 2019 to July 2020. In a previous detailed report [16], we described the findings of a longitudinal study of subclinical mastitis in sheep in Greece. During that work, four visits were made to 12 sheep farms throughout a milking period, starting immediately (within 5 days) after the removal of lambs from the dams and the subsequent start of milking of the ewes, and up to the end of the milking period, which allowed a total monitoring of 6 to 6.5 months, i.e., for the entirety of the milking period [16]. The findings of that study indicated that the incidence risk of subclinical mastitis was 52% and that the predominant causal agents were staphylococci, which accounted for over 90% of the causal pathogens identified in the study [16]. That publication focused on the clinical, aetiological and epidemiological features of mastitis. The increased frequency of isolation of staphylococci from cases of the infection indicates that there is merit in further studying the laboratory characteristics of these pathogens, in particular their antibiotic resistance patterns; this is important from a public health viewpoint (given that in Greece, sheep milk is widely used for manufacturing dairy products and genetic material of antibiotic-resistant staphylococci may thus be transferred to people) and from the viewpoint of therapeutics of the animals.

The present paper extends the previous findings and is complementary to the previous publication, focusing on the following laboratory characteristics of the staphylococcal isolates: antibiotic resistance and association with biofilm formation. The specific objectives of the present study were (a) to describe the incidence of isolation of antibiotic-resistant staphylococci from cases of mastitis throughout the milking period in dairy sheep flocks, i.e., in a longitudinal study, and (b) to identify relevant risk factors, which would contribute to the sustainable control of the infection.

## 2. Results

### 2.1. Descriptive Findings

Antibiotic resistance was detected in 57 of the 179 staphylococcal isolates recovered from cases of subclinical mastitis (31.8%; 95% confidence intervals (CIs): 25.5–39.0%). The median number (interquartile range) of antibiotics to which resistance was seen was 3 (2) per isolate.

Resistance was recorded against 11 antibiotics, most often against ampicillin (63.2% of resistant isolates), penicillin (63.2%) and tetracycline (47.4%) (Table 1). Isolates resistant to ampicillin and penicillin were recovered from all 12 farms; isolates resistant to erythromycin and tetracycline were recovered from 11 (91.7%) and 10 (83.3%) farms, respectively (Table 1). No isolates were found to be resistant to amikacin, fucidic acid, mupirocin, rifampicin, teicoplanin, trimethoprim–sulfamethoxazole and vancomycin.

Resistance was detected more frequently among coagulase-negative isolates (35.5%; 95% CI: 28.1–43.7%) than among *S. aureus* isolates (18.4%; 95% CI: 9.2–33.4%) (*p* = 0.045). Among coagulase-negative isolates, there was also resistance to more antibiotics than among *S. aureus* isolates: 3 (2) versus 2 (1), respectively (*p* = 0.019). For coagulase-negative species, of which over 10 isolates had been recovered and tested, the proportion of resistant isolates was 33.3% for *S. chromogenes*, 28.6% for *S. epidermidis*, 26.5% for *S. simulans* and 22.2% for *S. xylosus* (Tables S1 and S2).

Twenty-one multidrug-resistant isolates (11.7%; 95% CI: 7.8–17.3%) were recovered. All these isolates were coagulase-negative staphylococci (Table S1).

In five cases, the same bacteria (2 *S. aureus*, 2 *S. simulans*, 1 *S. xylosus*), also with identical antibiotic resistance profiles, were recovered from the two affected mammary glands of the same ewe. Moreover, in two cases, repeated isolation of staphylococci (1 *S. aureus*, 1 *S. simulans*), also with identical antibiotic resistance profiles, were recovered from consecutive samplings (1.5 months apart) from the same mammary gland of the same ewe.

### 2.2. Incidence Risk of Isolation of Resistant Staphylococci

Overall, the incidence risk of isolation of staphylococci resistant to at least one (any) antibiotic throughout the study period was 23.8% (95% CI: 18.8–29.5%) and varied between farms from 10.0% to 45.0% (Table S3). The incidence risk of isolation of staphylococci resistant to oxacillin was 5.0% (95% CI: 2.9–8.5%), varying from 0.0% to 10.0% between farms. Finally, the incidence risk of isolation of multidrug-resistant staphylococci was 8.8% (95% CI: 5.8% to 13.0%); it varied between farms from 0.0% to 20.0%. The estimated incidence risk of the above three outcomes in the study farms combined was 27.5% (95% CI: 25.8–29.2%), 6.1% (95% CI: 5.2–7.0%) and 11.1% (95% CI: 10.0–12.3%), respectively.

**Figure 1:**
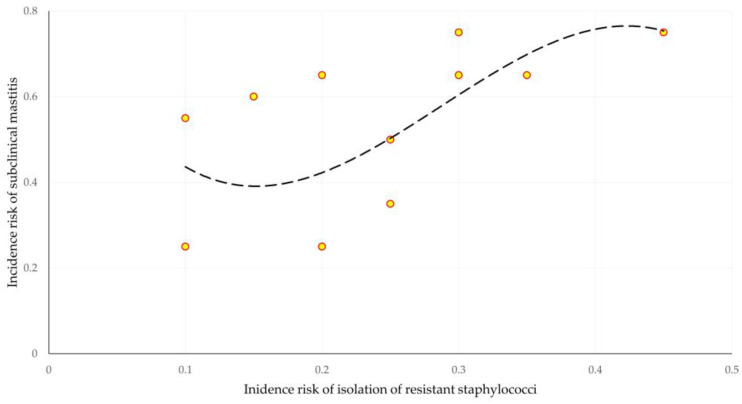
Cross-plot of incidence risk of subclinical mastitis versus incidence risk of isolation of staphylococci resistant to at least one (any) antibiotic (dashed line is trendline).

There was evidence of correlation between the incidence risk of subclinical mastitis and the incidence risk of isolation of staphylococci resistant to at least one (any) antibiotic (*r_sp_* = 0.649, *p* = 0.022) (Figure 1), as well as the incidence risk of isolation of staphylococci resistant to oxacillin (*r_sp_* = 0.631, *p* = 0.028). No correlation was seen between the incidence risk of subclinical mastitis and the incidence risk of isolation of multidrug-resistant staphylococci (*r_sp_* = 0.260, *p* = 0.42). There was no significant difference between the three correlation coefficients (*z* < 1.09, *p* > 0.28 for all comparisons).

**Figure 2:**
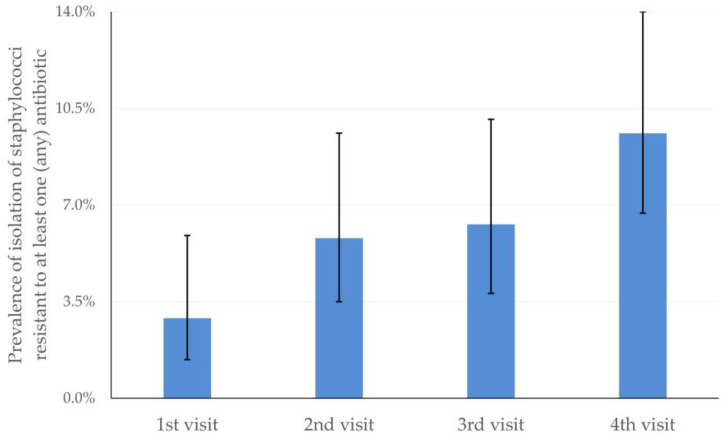
Progressive change in the prevalence of isolation of staphylococci resistant to at least one (any) antibiotic (bars indicate 95% confidence intervals).

The prevalence of isolation of staphylococci resistant to at least one (any) antibiotic progressively increased from 2.9% on the first to 9.6% on the fourth sampling (*p* = 0.026 between sampling occasions) (Figure 2). However, no significant change was seen in the prevalence of isolation of staphylococci resistant to oxacillin (*p* = 0.57) and the prevalence of isolation of multidrug-resistant staphylococci (*p* = 0.19).

Finally, some tendency for correlation was seen between the incidence risk of isolation of staphylococci resistant to at least one (any) antibiotic and the cases of recurrence of subclinical mastitis recorded in the same flock during the study period (*r_sp_* = 0.519, *p* = 0.08).

### 2.3. Risk Factors

The results of the univariable analysis of correlations between the incidence risk of isolation of antibiotic-resistant staphylococci and the various parameters studied are detailed in Table S4.

**Figure 3:**
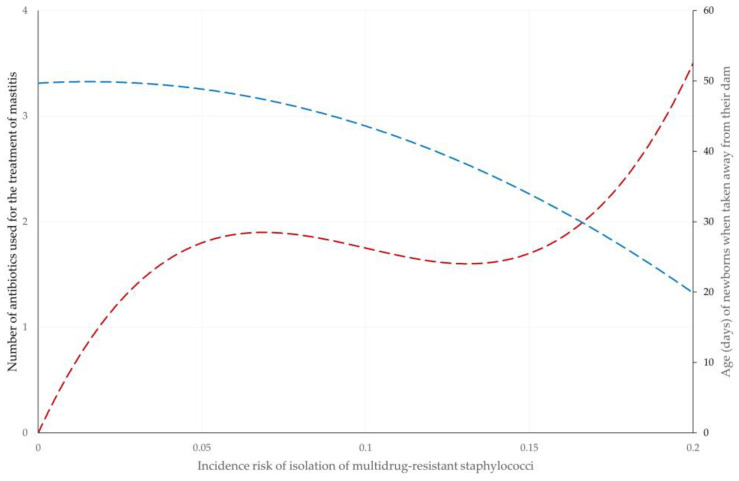
Trendlines for association of the incidence risk of isolation of multidrug-resistant staphylococci throughout a milking period with the number of antibiotics used on the farm for the treatment of mastitis (dark red) and the age of newborns when taken away from the dam (blue).

With regard to increased incidence risk of isolation of staphylococci resistant to at least one (any) antibiotic and increased incidence risk of isolation of staphylococci resistant to oxacillin, only the omission of anti-staphylococcal mastitis vaccination of ewes (*p* = 0.004 and 0.017, respectively) emerged as significant. With regard to increased incidence risk of isolation of multidrug-resistant staphylococci, the following variables emerged as significant: (a) higher number of antibiotics used on the farm for the treatment of mastitis (*p* = 0.0008) and (b) younger age when lambs were taken away from their dam (*p* = 0.004) (Figure 3 and Figure S1). Details are in Table 2.

### 2.4. Associations with Biofilm Formation by Staphylococcal Isolates

Of the 179 staphylococcal isolates recovered from subclinical mastitis and assessed during this study, 117 (65.4%) were found to be biofilm-forming. There was a significant difference between the various staphylococcal species in the proportion of biofilm-forming isolates identified (*p* = 0.017). The highest proportions of biofilm-forming isolates were identified among *S. saprophyticus* (100.0% of isolates), *S. aureus* (84.2%) and *S. hominis* (80.0%) isolates (Table S5). Biofilm-forming isolates were recovered more frequently from flocks that had not been vaccinated against staphylococcal mastitis: 84.1% (53/63) of isolates recovered from these flocks versus 55.2% (64/116) of isolates from non-vaccinated flocks (*p* = 0.0001).

**Figure 4:**
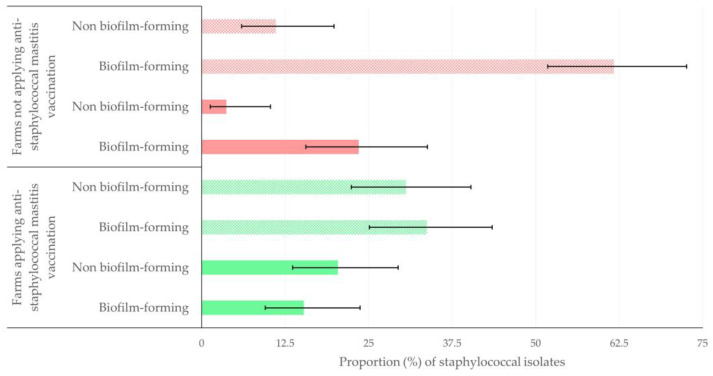
Proportion (%) (error bars: 95% confidence intervals) of biofilm-forming or non-biofilm-forming staphylococci isolated from sheep farms on which vaccination against staphylococcal was (green bars) or was not (red bars) applied; solid bars indicate antibiotic-resistant isolates and motif bars indicate non-antibiotic-resistant isolates.

There was no overall difference in the proportion of resistant isolates found among biofilm-forming (34/117 = 29.1%) or non-biofilm-forming (23/62 = 37.1%) staphylococci (*p* = 0.27). However, most biofilm-forming antibiotic-resistant staphylococci were recovered from farms where anti-staphylococcal mastitis vaccination was not applied: 19/34 isolates (55.9%) versus 15/34 antibiotic-resistant isolates (44.1%) from farms where anti-staphylococcal mastitis vaccination was applied. In the latter farms, most antibiotic-resistant isolates were not biofilm-forming: 20/23 (87.0%) and 3/20 (13.0%), respectively (*p* = 0.001) (Figure 4, Table S6).

## 3. Discussion

### 3.1. Antibiotic Resistance of Staphylococcal Isolates

This study provides a detailed account of the patterns of isolation of antibiotic-resistant staphylococci from ovine mastitis throughout the milking period.

Effective treatment of mastitis requires the combination of speed and efficacy. Treatment should be instigated immediately after diagnosis of the initial signs of the infections and should be carried out with effective antimicrobial agents. Administration of the appropriate drug should be performed following the identification of causal pathogen(s) and the establishment of its (their) antibiotic susceptibility profile. Usage of narrow-spectrum antibiotics should be prioritized, as it contributes to maintaining antibiotic efficacy against the causal agents.

The present paper extends previous findings, focusing on the following laboratory characteristics of the staphylococcal isolates: antibiotic resistance and association with biofilm formation.

The present results are within the general pattern of antibiotic resistance of staphylococcal isolates from cases of mastitis in sheep, as described previously [3,4,5,6,7,8,9,10]. In this respect, the differences between these resistance rates and findings in countries outside Europe are notable; for example, reports of a high rate of resistance to amoxicillin, erythromycin, lincomycin, streptomycin and tetracycline (>35% of staphylococci evaluated) in Brazil [18], to penicillin and tetracycline (>55% of *S. aureus* evaluated) in Iran [19] or to ampicillin, oxacillin and tetracycline (>45% of *S. aureus* evaluated) in Kenya [20] have been published. These differences may be the result of the strict policies regarding prescription of veterinary drugs applied in the European Union, coupled with the continuous campaigning against inappropriate antibiotic dispensation and usage.

The benefits of these policies can be seen when considering the findings of a study on antibiotic resistance of staphylococci from cases of ovine mastitis, performed in Greece over 25 years ago, in which the resistance rates of *S. aureus* to antibiotics were higher than those of coagulase-negative staphylococci [21], a feature that has since been reversed. Hence, sustained campaigns for appropriate antibiotic usage in veterinary work appear to have tangible benefits.

The present findings indicate that, in sheep, throughout this milking period, approximately one-third of the staphylococcal isolates causing mastitis were antibiotic-resistant. In line with the findings of other researchers [3,4,5,6,7,8,9,10], limited antibiotic resistance was seen among *S. aureus* isolates, whilst a higher proportion of coagulase-negative isolates were found to be resistant. Coagulase-negative isolates are the primary cause of subclinical mastitis, which requires specific laboratory tests for diagnosis and thus most often remains undiagnosed and untreated. Thus, causal bacteria are present in the animals on the farm and can be exposed to antibiotics administered to animals for other reasons, which may contribute to the development of resistance. Further, some coagulase-negative species (e.g., *S. haemolyticus*, *S. hominis*, *S. saprophyticus*) mainly associated with human infections [22], and recovered from cases of mastitis and found to be resistant in the present study, might have originated from people (e.g., farmers, farm staff, visitors), whence they would have been transmitted to the animal population.

The finding of resistance to fosfomycin of some isolates lends support to the above hypothesis. In Greece, fosfomycin is used only for treatment of human infections; the antibiotic is not licensed for veterinary use. Therefore, it may be postulated that detection of fosfomycin resistance indicates that relevant isolates were of human origin.

In a recent, extensive field investigation performed in Greece, it was revealed, through information obtained from sheep farmers across the country, that the antibiotics most frequently used for the treatment of clinical mastitis were penicillin (81% of farms) and oxytetracycline (22% of farms) [2]. In most cases (88.9% of farms), treatment of clinical mastitis was carried out by administering antibiotics in injectable forms rather than by the intramammary route [2]. In cattle herds, it was found that the systematic administration of antibiotics for the treatment of mastitis was associated with a more frequent isolation of multidrug-resistant staphylococci in comparison to using the intra-mammary products [23].

### 3.2. Risk Factors for High Incidence of Antibiotic Resistance

The significant correlation between the incidence risk of subclinical mastitis and the incidence risk of isolation of resistant staphylococci confirms that the best means to avoid emergence of antibiotic-resistant isolates would be to prevent the infection. Controlling mastitis on sheep farms is important for improving the welfare of the animals on the farms [24], as well as for increasing the profitability of farms. Additionally, this would also contribute to reducing the spread of antibiotic-resistant bacterial isolates.

This can be seen in the identification of vaccination against anti-staphylococcal mastitis as a risk factor for lower incidence risk of isolation of antibiotic-resistant staphylococci. There are two possible reasons for this. First, anti-staphylococcal mastitis vaccination has been documented to decrease the incidence risk of staphylococcal mastitis in sheep [25], this being the major reason for the emergence of vaccination as a risk factor in the present study. A decrease in cases of the infection subsequent to vaccination reduces the chance for pathogen mutation into an antibiotic resistance form [26]. Moreover, the utilization, as the antigenic component of anti-staphylococcal vaccines, of extracellular proteins present in the biofilm matrix of *S. aureus* can reduce the number of bacterial cells present within the biofilm and thus contribute to reducing the chances for mutation to develop resistance in a model of mesh-associated infection [27,28].

Assessment of resistance to oxacillin is used to detect staphylococcal isolates with resistance to methicillin (Methicillin-Resistant Staphylococci), which corresponds to resistance to all *β*-lactams. In the present study, whilst resistance to oxacillin was detected, all such isolates were nevertheless sensitive to vancomycin (an antibiotic that may be used as an alternative therapeutic agent).

However, for the isolation of multidrug-resistant staphylococci, other health management factors emerged as significant. The use of a higher number of antibiotics in the treatment of clinical mastitis contributes to the development of multidrug resistance in staphylococcal isolates in the farms. Overuse of antibiotics is a universal risk factor for the development of antibiotic resistance [29,30], and in this case, it is a risk factor for the isolation of multidrug-resistant staphylococci. Further, the identification of earlier age of lambs taken away from their dam as another risk factor can possibly be associated with the increased incidence of health problems, including various infections, seen in such management systems [31,32]. Thus, there would be an increased need for antibiotic usage in such farms to treat lamb infections, which can contribute to the development of multidrug resistance to staphylococcal isolates prevalent on the farms.

The knowledge of risk factors for the development of antibiotic resistance by staphylococci from cases of mastitis can contribute to prevention of the problem. In Greece, dairy products from ewes’ milk (cheese, yoghurt) are important agricultural products consumed widely, as well as accounting for a significant share of relevant exports. Therefore, there is interest from a public health viewpoint to minimize the presence of antibiotic-resistant bacteria in milk from sheep.

## 4. Materials and Methods

### 4.1. Field Work

Twelve dairy, machine-milked sheep flocks were visited repeatedly (four times throughout an entire milking period) for collection of milk samples and for obtaining relevant information (Table S7). During the visits, samples were collected from both mammary glands of 20 ewes on each farm. The full details regarding the survey work, i.e., collection of information about management in the farm, selection and inclusion and clinical examination of animals, sampling procedures applied in the farms and handling of samples, have been described by Michael et al. [16].

### 4.2. Laboratory Work

Cytological examination of milk samples obtained included the California Mastitis Test and Giemsa staining [16].

Primary cultures were performed by conventional bacteriological techniques, using Columbia blood agar plates (BioPrepare Microbiology, Athens, Greece) and staphylococcus selective medium (mannitol salt agar; BioPrepare Microbiology), and initial bacterial identification of staphylococci was performed by applying standard methods [33,34]. The staphylococcal isolates recovered were identified to species level by means of MALDI-TOF MS (VITEK MS; BioMerieux, Marcy-l’-Étoile, France).

Subsequently, the staphylococcal isolates obtained in this study were assessed for sensitivity to antibiotics. In total, 179 staphylococcal isolates recovered from cases of subclinical mastitis during this study were evaluated. Susceptibility/resistance to the following 18 antibiotics was tested: amikacin, ampicillin, ciprofloxacin, clindamycin, erythromycin, fosfomycin, fusidic acid, gentamicin, moxifloxacin, mupirocin, oxacillin, penicillin G, rifampicin, teicoplanin, tetracycline, tobramycin, trimethoprim–sulfamethoxazole and vancomycin (Table S8). Testing was carried out by means of the automated system BD Phoenix™ M50 (BD Diagnostic Systems, Sparks, MD, USA). For the interpretation, the results of the antibiotic resistance testing were compared to the respective breakpoints for the various staphylococcal species in accordance with the recommendations of the European Committee on Antimicrobial Susceptibility Testing (www.eucast.org).

Finally, for the assessment of the formation of biofilm under in vitro conditions, the results of (a) culture appearance on Congo red agar plates and (b) micro-plate adhesion test were combined by performing these techniques as previously detailed by Vasileiou [25].

### 4.3. Data Management and Analysis

#### 4.3.1. Data Management

The definition of subclinical mastitis was based on a combination of bacteriological and cytological findings, always in the absence of clinically evident findings as defined previously (Table S9). Staphylococcal subclinical mastitis was defined as when *Staphylococcus* spp. was identified as the causal pathogen of subclinical mastitis.

The outcomes of ‘isolation of staphylococci from cases of subclinical mastitis, resistant to at least one (any) antibiotic’, ‘isolation of oxacillin-resistant staphylococci from cases of subclinical mastitis’ and ‘isolation of multidrug-resistant staphylococci from cases of subclinical mastitis’ were considered. All the above outcomes referred to ewes. Hence, animals from both mammary glands of which antibiotic-resistant staphylococci were isolated were considered as one case.

Incidence risk for each of the above outcomes was defined as the proportion of animals at risk that developed the condition when the time at risk differed between animals (i.e., during the entire milking period, which varied between individuals) [25]. Animals that cleared the infection (i.e., were found without subclinical mastitis) were considered at risk at subsequent visits; if they developed the infection again during the study period, they were still counted as one case.

For characterization of biofilm formation by staphylococcal isolates, the results of the two methods that had been employed were combined [25], and the isolates were characterized as biofilm-forming or non-biofilm-forming.

The interpretation of the results of testing for antibiotic resistance was based on the criteria of the European Committee on Antimicrobial Susceptibility Testing (EUCAST) (http://www.eucast.org). Isolates found to be resistant to at least three different classes of antibiotics were classified as multidrug-resistant isolates [35]. Antibiotics were categorised according to the classification of the European Medicines Agency [17] into four categories: A, B, C, D, respectively, corresponding to use with ‘avoidance’, ‘restriction’, ‘caution’ and ‘prudence’ [17].

#### 4.3.2. Statistical Analysis

All data were entered into Microsoft Excel and analysed using IBM SPSS Statistics (ver. 21) (IBM; Armonk, NY, USA). Basic descriptive analysis was performed. Exact binomial confidence intervals (CIs) were obtained.

Frequencies were compared by using Pearson’s chi-square test or Fisher’s exact test, as appropriate. The Mann–Whitney test was employed to compare groups. Correlation analysis was performed by means of Spearman’s rank correlation.

Potential risk factors for isolation of antibiotic-resistant staphylococci, as specified in the above-described three outcomes, were considered. In total, 20 parameters (Table S10) were evaluated for potential association with each of the three outcomes. For each of these 20 parameters, results were taken directly from the answers obtained during the interview or calculated based on these answers. Initially, in univariable analyses, the importance of risk factors was evaluated by using Spearman’s rank correlation between the incidence risk of each of the above outcomes and the results of the various parameters (*n* = 20) assessed. Then, a multivariable model was developed for each of the above three outcomes; parameters found with *p* < 0.2 in the preceding univariable analyses were offered to this model. Progressively, variables entered into the multivariable model were removed from the model by using backwards elimination. The likelihood ratio test was performed to assess the *p*-value of each parameter; among those found with *p* ≥ 0.2, the one with the largest *p* was removed from the model. The procedure was repeated until no variable with *p* ≥ 0.2 could be removed from the model. The variables included in the final multivariable model constructed for each outcome are detailed in Table S11.

In all analyses, statistical significance was defined at *p* < 0.05.

## 5. Conclusions

This study referred to a longitudinal investigation into the incidence risk of isolation of antibiotic-resistant staphylococci from cases of mastitis in dairy ewes. The incidence risk of isolation of staphylococci resistant to at least one (any) antibiotic throughout a milking period was 23.8%. There was a progressive increase in the frequency of isolation of resistant staphylococcal isolates, in line with the progressively increasing frequency of subclinical mastitis. Antibiotic resistance was detected in approximately in one-third of the isolates studied against 11 antibiotics. Omission of vaccination against anti-staphylococcal mastitis was found to be a risk factor for increased incidence risk of isolation of resistant staphylococci, with most biofilm-forming resistant staphylococci recovered from farms where anti-staphylococcal mastitis vaccination was not applied.

## Figures and Tables

**Table 1:** Frequency of resistance of staphylococcal isolates from cases of subclinical mastitis to 17 antibiotics.

Antibiotic	Antibiotic Category ^1^	No. of Resistant Isolates (Proportion among Resistant Isolates)	No. of Farms Where Resistant Isolates Were Recovered (Proportion among Farms in This Study)
Ampicillin	D	36 (63.2%)	12 (100.0%)
Ciprofloxacin	B	1 (1.8%)	1 (8.3%)
Clindamycin	C	20 (35.1%)	9 (75.0%)
Erythromycin	C	20 (35.1%)	11 (91.7%)
Fosfomycin	A	11 (19.3%)	8 (66.7%)
Gentamicin	C	4 (7.0%)	4 (44.4%)
Morifloxacin	B	1 (1.8%)	1 (8.3%)
Oxacillin	D	12 (21.1%)	8 (75.0%)
Penicillin G	D	36 (63.2%)	12 (100.0%)
Tetracycline	D	27 (47.4%)	10 (83.3%)
Tobramycin	C	1 (1.8%)	1 (8.3%)

^1^ Antibiotic category according to European Medicines Agency [17] for use in animals.

**Table 2:** Results of multivariable analysis for variables with a significant association with increased incidence risk of isolation of antibiotic-resistant staphylococci from cases of subclinical mastitis in sheep.

**Isolation of Staphylococci Resistant to at Least One (Any) Antibiotic**		
Variable	Odds Ratio ^1^ (95% CI ^2^)	*p*
Application of anti-staphylococcal mastitis vaccination		0.004
No	2.399 (1.262–4.557)	0.008
Yes	reference	-
**Isolation of Staphylococci Resistant to Oxacillin**		
Variable	Odds Ratio (95% CI) ^1^	*p*
Application of anti-staphylococcal mastitis vaccination		0.017
No	3.222 (0.998–10.404)	0.05
Yes	reference ^1^	-
**Isolation of Multidrug-Resistant Staphylococci**		
Variable	Odds Risk (±standard error)	*p*
Number of antibiotics used for the treatment of mastitis		0.0008
Per unit increase	1.038 ± 1.007	0.0008
Age (days) of newborns when taken away from the dam		0.004
Per day decrease	0.998 ± 1.001	0.004

^1^ Odds ratio calculated against the lowest prevalence associations of variables (reference); ^2^ CI: confidence intervals.

## Data Availability

Most data presented in this study are in the Supplementary Materials. The remaining data are available on request from the corresponding author. The data are not publicly available as they form part of the Ph.D. thesis of the first author, which has not yet been examined, approved, and uploaded in the official depository of Ph.D. theses from Greek Universities.

## Supplementary Materials

The following supporting information can be downloaded at: https://www.mdpi.com/article/10.3390/antibiotics12121703/s1, Table S1: Frequency of resistant isolates among different staphylococcal species recovered from cases of subclinical mastitis during this longitudinal study throughout a milking period in 12 sheep flocks in Greece. Table S2: Frequency of susceptibility/resistance to individual antibiotics of staphylococcal isolates recovered from cases of subclinical mastitis during this longitudinal study throughout a milking period in 12 sheep flocks in Greece; Table S3: Incidence risk of isolation of antibiotic-resistant staphylococci from cases of subclinical mastitis among 12 sheep flocks in Greece monitored throughout a milking period; Table S4: Results of univariable analysis of variables evaluated for association with the outcomes of ‘isolation of staphylococci from cases of subclinical mastitis, resistant to at least one (any) antibiotic’, ‘isolation of oxacillin-resistant staphylococci from cases of subclinical mastitis’ and ‘isolation of multidrug-resistant staphylococci from cases of subclinical mastitis’ recovered from cases of subclinical mastitis during this longitudinal study throughout a milking period in 12 sheep flocks in Greece; Figure S1: Scatter plot of incidence of isolation of multidrug-resistant staphylococci from cases of subclinical mastitis during this longitudinal study throughout a milking period in 12 sheep flocks in Greece and age of newborns when taken away from the dam; Table S5: Results of biofilm formation by staphylococcal isolates from cases of subclinical mastitis among 12 sheep flocks in Greece monitored throughout a milking period; Table S6: Contingency table indicating associations between anti-staphylococcal mastitis vaccination status and biofilm formation, according to antibiotic resistance, of staphylococcal isolates recovered from subclinical mastitis among 12 sheep flocks in Greece monitored throughout a milking period. Table S7: Details of 12 flocks included in this longitudinal study of subclinical mastitis in Greece; Table S8: Concentrations of antibiotics against which resistance of staphylococcal isolates was tested in the automated system BD Phoenix™ M50; Table S9: Detailed description of the criteria for definition of subclinical mastitis in sheep flocks; Table S10: Variables evaluated for potential association with the isolation of antibiotic-resistant staphylococci from cases of subclinical mastitis during this longitudinal study throughout a milking period in 12 sheep flocks in Greece; Table S11: Details of multivariable models employed for the evaluation of risk factors for the isolation of antibiotic-resistant staphylococci from cases of subclinical mastitis during this longitudinal study throughout a milking period in 12 sheep flocks in Greece.

## References

[1] Gelasakis A.I., Mavrogianni V.S., Petridis I.G., Vasileiou N.G.C., Fthenakis G.C. (2015). Mastitis in sheep—The last 10 years and the future of research. Vet. Microbiol..

[2] Lianou D.T. (2023). Mapping the Small Ruminant Industry in Greece: Health Management and Diseases of Animals, Preventive Veterinary Medicine and Therapeutics, Reproductive Performance, Production Outcomes, Veterinary Public Health, Socio-demographic Characteristics of the Farmers. Ph.D. Thesis.

[3] Vautor E., Carsenti-Dellamonica H., Sabah M., Mancini G., Pepin M., Dellamonica P. (2007). Characterization of Staphylococcus aureus isolates recovered from dairy sheep farms (agr group, adherence, slime, resistance to antibiotics). Small Rumin. Res..

[4] Onni T., Sanna G., Larsen J., Tola S. (2011). Antimicrobial susceptibilities and population structure of *Staphylococcus epidermidis* associated with ovine mastitis. Vet. Microbiol..

[5] Ergun Y., Aslantas O., Kirecci E., Ozturk F., Ceylan A., Boyar Y. (2012). Antimicrobial susceptibility, presence of resistance genes and biofilm formation in coagulase negative staphylococci isolated from subclinical sheep mastitis. Kafkas Univ. Vet. Fakult. Derg..

[6] Unal N., Askar S., Macun H.C., Sakarya F., Altun B., Yildirim M. (2012). Panton-Valentine leukocidin and some exotoxins of Staphylococcus aureus and antimicrobial susceptibility profiles of staphylococci isolated from milks of small ruminants. Trop. Anim. Health Prod..

[7] Martins K.B., Faccioli P.Y., Bonesso M.F., Fernandes S., Oliveira A.A., Dantas A., Zafalon L.F., Cunha M.D.R.S. (2017). Characteristics of resistance and virulence factors in different species of coagulase-negative staphylococci isolated from milk of healthy sheep and animals with subclinical mastitis. J. Dairy Sci..

[8] Abdalhamed A.M., Zeedan G.S.G., Abou Zeina H.A.A. (2018). Isolation and identification of bacteria causing mastitis in small ruminants and their susceptibility to antibiotics, honey, essential oils, and plant extracts. Vet. World.

[9] Azzi O., Lai F., Tennah S., Menoueri M.N., Achek R., Azara E., Tola S. (2020). Spa-typing and antimicrobial susceptibility of *Staphylococcus aureus* isolated from clinical sheep mastitis in Medea province, Algeria. Small Rumin. Res..

[10] Andrade N.C., Laranjo M., Costa M.M., Queiroga M.C. (2021). Virulence factors in staphylococcus associated with small ruminant mastitis: Biofilm production and antimicrobial resistance genes. Antibiotics.

[11] Azara C., Longheu G., Sanna G., Tola S. (2017). Biofilm formation and virulence factor analysis of *Staphylococcus aureus* isolates collected from ovine mastitis. J. Appl. Microbiol..

[12] Regecová I., Vyrostková J., Zigo F., Gregová G., Kovácová M. (2021). Detection of antimicrobial resistance of bacteria *Staphylococcus chromogenes* isolated from sheep’s milk and cheese. Antibiotics.

[13] Todhunter D.A., Cantwell L.L., Smith K.L., Hoblet K.H., Hogan J.S. (1993). Characteristics of coagulase-negative staphylococci isolated from bovine intramammary infections. Vet. Microbiol..

[14] Sun M., Gao J., Ali T., Yu D., Zhang S.Y., Khan S.U., Fanning S., Han B. (2017). Characteristics of *Aerococcus viridans* isolated from bovine subclinical mastitis and its effect on milk SCC, yield, and composition. Trop. Anim. Health Prod..

[15] Singha S., Koop G., Persson Y., Hossain D., Scanlon L., Derks M., Hoque M.A., Rahman M.M. (2021). Incidence, etiology, and risk factors of clinical mastitis in dairy cows under semi-tropical circumstances in Chattogram, Bangladesh. Animals.

[16] Michael C.K., Lianou D.T., Vasileiou N.G.C., Mavrogianni V.S., Petinaki E., Fthenakis G.C. (2023). Longitudinal study of subclinical mastitis in sheep in Greece: An investigation into incidence risk, associations with milk quality and risk factors of the infection. Animals.

[17] European Medicines Agency (2019). Categorisation of Antibiotics in the European Union.

[18] Franca C.A., Peixoto R.M., Cavalcante M.B., Melo N.F., Oliveira C.J.B., Veschi J.A., Mota R.A., Costa M.M. (2012). Antimicrobial resistance of *Staphylococcus* spp. from small ruminant mastitis in Brazil. Pesq. Vet. Bras..

[19] Jamali H., Paydar M., Radmehr B., Ismail S., Dadrasnia A. (2015). Prevalence and antimicrobial resistance of Staphylococcus aureus isolated from raw milk and dairy products. Food Control.

[20] Omwenga I., Aboge G.O., Mitema E.S., Obiero G., Ngaywa C., Ngwili N., Wamwere G., Wainaina M., Bett B. (2021). Antimicrobial usage and detection of multidrug-resistant *Staphylococcus aureus*, including methicillin-resistant strains in raw milk of livestock from Northern Kenya. Microb. Drug Res..

[21] Fthenakis G.C. (1998). Susceptibility to antibiotics of staphylococcal isolates from cases of ovine or bovine mastitis in Greece. Small Rumin. Res..

[22] Bartlett A., Padfield D., Lear L., Bendall R., Vos M. (2022). A comprehensive list of bacterial pathogens infecting humans. Microbiology.

[23] Nobrega D.B., De Buck J., Barkema H.W. (2018). Antimicrobial resistance in non-aureus staphylococci isolated from milk is associated with systemic but not intramammary administration of antimicrobials in dairy cattle. J. Dairy Sci..

[24] European Food Safety Authority (2014). Scientific opinion on the welfare risks related to the farming of sheep for wool, meat and milk production. EFSA J..

[25] Vasileiou N.G.C. (2019). Mastitis in Ewes Associated with *Staphylococcus* spp.: New Clinical, Epidemiological, Management, Microbiological, and Zoonotic Findings and Evaluation of a Novel Vaccine Against the Disease. Ph.D. Thesis.

[26] Atkins K.E., Lafferty E.I., Deeny S.R., Davies N.G., Robotham J.V., Jit M. (2018). Use of mathematical modelling to assess the impact of vaccines on antibiotic resistance. Lancet Infect. Dis..

[27] Gil C., Solano C., Burgui S., Latasa C., García B., Toledo-Arana A., Lasa I., Valle J. (2014). Biofilm matrix exoproteins induce a protective immune response against Staphylococcus aureus biofilm infection. Infect. Immun..

[28] Flores-Valdez M.A. (2016). Vaccines directed against microorganisms or their products present during biofilm lifestyle: Can we make a translation as a broad biological model to tuberculosis?. Front. Microbiol..

[29] Ventola C.L. (2015). The antibiotic resistance crisis: Part 1: Causes and threats. Pharm. Ther..

[30] Byrne M.K., Miellet S., McGlinn A., Fish J., Meedya S., Reynolds N., van Oijen A.M. (2019). The drivers of antibiotic use and misuse: The development and investigation of a theory driven community measure. BMC Public Health.

[31] Campbell B.J., Pullin A.N., Pairis-Garcia M.D., McCutcheon J.S., Lowe G.D., Campler M.R., Fluharty F.L. (2017). The effects of alternative weaning strategies on lamb health and performance. Small Rumin. Res..

[32] Freitas-de-Melo A., Orihuela A., Hötzel M.J., Ungerfeld R. (2022). What do we know and need to know about weaning in sheep? An overview of weaning practises, stress and welfare. Front. Anim. Sci..

[33] Barrow G.I., Feltham R.K.A. (1993). Manual for the Identification of Medical Bacteria.

[34] Euzeby J.P. (1997). List of bacterial names with standing in nomenclature: A folder available on the Internet. Int. J. Syst. Bacteriol..

[35] Magiorakos A.P., Srinivasan A., Carey R.B., Carmeli Y., Falagas M.E., Giske C.G., Harbarth S., Hindler J.F., Kahlmeter G., Olsson-Liljequist B. (2012). Multidrug-resistant, extensively drug-resistant and pandrug-resistant bacteria: An international expert proposal for interim standard definitions for acquired resistance. Clin. Microbiol. Infect..

