# Supplementary material for: Longitudinal Study of Antibiotic Resistance of Staphylococci from Cases of Subclinical Mastitis in Sheep in Greece: Incidence and Risk Factors
Source: Antibiotics (Basel). 2023 Dec 7;12(12):1703. doi: 10.3390/antibiotics12121703 (PMC10741230; doi:10.3390/antibiotics12121703)
Supplement: Supplementary file 1 [file antibiotics-12-01703-s001.zip › antibiotics-2746817-SI.pdf]

Longitudinal Study of Antibiotic Resistance of Staphylococci from Cases of Subclinical Mastitis in Sheep in Greece: Incidence and Risk Factors

Charalambia K. Michael, Daphne T. Lianou, Katerina Tsilipounidaki, Zoe Florou, Natalia G.C. Vasileiou, Vasia S. Mavrogianni, Efthymia Petinaki and George C. Fthenakis

Table S1. Frequency of resistant isolates among different staphylococcal species recovered from cases of subclinical mastitis during this longitudinal study throughout a milking period in 12 sheep flocks in Greece.

Staphylococcal Species	Resistant Isolates	Multidrug-Resistant Isolates
S. aureus (*n* = 38 [1])	7 (18.4% [2])	0 (0.0% [2])
S. capitis (*n* = 2)	1 (50.0%)	0 (0.0%)
S. caprae (*n* = 6)	1 (16.7%)	1 (16.7%)
S. chromogenes (*n* = 24)	8 (33.3%)	4 (16.7%)
S. epidermidis (*n* = 28)	8 (28.6%)	3 (10.7%)
S. equorum (*n* = 4)	3 (75.0%)	2 (50.0%)
S. haemolyticus (*n* = 4)	3 (75.0%)	1 (25.0%)
S. hominis (*n* = 5)	4 (80.0%)	2 (40.0%)
S. lentus (*n* = 9)	5 (55.6%)	1 (11.1%)
S. saprophyticus (*n* = 1)	1 (100.0%)	1 (100.0%)
S. schleiferi (*n* = 1)	0 (0.0%)	0 (0.0%)
S. sciuri (*n* = 3)	1 (33.3%)	1 (33.3%)
S. simulans (*n* = 34)	9 (26.5%)	4 (11.8%)
S. warneri (*n* = 2)	2 (100.0%)	0 (0.0%)
S. xylosus (*n* = 18)	4 (22.2%)	1 (5.6%)
All staphylococcal species (n = 179)	57 (31.8%)	21 (11.7%)

[1] total no. of isolates recovered and tested; [2] proportion of isolates resistant to at least one (any) antibiotic or of multidrug-resistant isolates among all isolates of that species.

Table S2. Frequency of susceptibility / resistance to individual antibiotics of staphylococcal isolates recovered from cases of subclinical mastitis during this longitudinal study throughout a milking period in 12 sheep flocks in Greece.

	n [1]	AMI [2]	AMP	CIP	CLI	ERY	FOS	FUS	GEN	MOX	MUP	OXA	PEN	RIF	TEI	TET	TOB	SXT	VAN
S. aureus	7		4		1	1						2	4			2			
S. capitis	1						1									1			
S. caprae	1		1		1								1			1			
S. chromogenes	8		6		1	3	1		1			2	6			5	1		
S. epidermidis	8		5		3	3	2		1			2	5			5			
S. equorum	3		3		2	2	1					1	3						
S. haemolyticus	3		1		2	1	1					1	1			1			
S. hominis	4		3		1	1	2					2	3			1			
S. lentus	5		3		2	2	1		1				3			2			
S. saprophyticus	1		1									1	1			1			
S. schleiferi	0																		
S. sciuri	1		1	1	1					1			1						
S. simulans	9		6		3	4						1	6			6			
S. warneri	2				1	1										1			
S. xylosus	4		2		2	2	2		1				2			1			
Total	57	0	36	1	20	20	11	0	4	1	0	12	36	0	0	27	1	0	0

[1] Total no. of isolates found with resistance to at least one (any) antibiotic).

[2] AMI: amikacin, AMP: ampicillin, CIP: ciprofloxacin, CLI: clindamycin, ERY: erythromycin, FOS: fosfomycin, FUS: fucidic acid, GEN: gentamicin, MOX: moxifloxacin, MUP: mupirocin, OXA: oxaxillin, PEN: penicillin, RIF: rifampicin, TEI: teicoplanin, TET: tetracycline, TOB: tobramycin, SXT: trimethoprim-sulfamethoxazole, VAN; vancomycin.

Table S3. Incidence risk of isolation of antibiotic-resistant staphylococci from cases of subclinical mastitis among 12 sheep flocks in Greece monitored throughout a milking period.

Farm	Incidence risk of isolation of oxacillin-resistant staphylococci	Incidence risk of isolation of staphylococci resistant to at least one (any) antibiotic)	Incidence risk of isolation of multidrug-resistant staphylococci
1	0.0%	25.0%	20.0%
2	5.0%	20.0%	5.0%
3	5.0%	25.0%	5.0%
4	5.0%	10.0%	5.0%
5	5.0%	20.0%	10.0%
6	10.0%	30.0%	10.0%
7	10.0%	35.0%	10.0%
8	0.0%	10.0%	5.0%
9	10.0%	45.0%	20.0%
10	0.0%	20.0%	5.0%
11	0.0%	15.0%	10.0%
12	10.0%	30.0%	0.0%

Table S4. Results (Spearman's rank correlation coefficients) of univariable analysis of variables (n = 20) evaluated for association with the outcomes of 'isolation of staphylococci from cases of subclinical mastitis, resistant to at least one (any) antibiotic', 'isolation of oxacillin-resistant staphylococci from cases of subclinical mastitis' and 'isolation of multidrug-resistant staphylococci from cases of subclinical mastitis' recovered from cases of subclinical mastitis during this longitudinal study throughout a milking period in 12 sheep flocks in Greece.

	**Isolation of staphylococci from cases of subclinical mastitis, resistant to at least one (any) antibiotic**		**Isolation of oxacillin-resistant staphylococci from cases of sub-clinical mastitis**		**Isolation of multidrug-resistant staphylococci from cases of subclinical mastitis**	
**Variables**	Correlation coefficient (r_{sp})	p-value	Correlation coefficient (r_{sp})	p-value	Correlation coefficient (r_{sp})	p-value
Management system applied in the flocks	0.002	0.99	–0.317	0.32	–0.094	0.77
No. of ewes in the flock	0.321	0.31	0.385	0.22	0.351	0.26
Average age of culling females	–0.383	0.22	0.124	0.70	–0.365	0.24
Month of the start of the lambing season	–0.026	0.94	0.311	0.32	–0.421	0.17
Total visits made annually by veterinarians to the flock during the preceding season	0.002	0.99	–0.195	0.54	0.079	0.81
Use of laboratory diagnostic examinations in samples of milk preventively	–0.124	0.70	–0.207	0.52	0.052	0.87
Age of lamb removal from their dams	–0.487	0.11	–0.376	0.23	–0.549	0.06
Daily number of milking sessions	0.480	0.11	0.236	0.46	0.266	0.40
Duration of the dry-period	–0.228	0.48	0.094	0.77	–0.085	0.79
Administration of selenium to pregnant ewes	0.394	0.21	0.548	0.07	–0.240	0.45
Administration of 'dry-ewe' treatment at the end of the lactation period	–0.073	0.82	0.2041	0.52	–0.460	0.13
Use of teat disinfection after milking	–0.033	0.92	0.195	0.54	–0.172	0.59
Number of antibiotics used for treatment of mastitis	0.022	0.95	–0.108	0.74	0.593	0.042
Route for administration of antimicrobials	–0.114	0.72	–0.195	0.54	0.402	0.19
Vaccination against staphylococcal mastitis	0.734	0.007	0.707	0.010	0.089	0.78

Annual frequency of systemic disinfections in the farm	0.125	0.70	–0.276	0.38	0.317	0.31
Temperature of cleaning water in the milking parlour	0.117	0.72	0.336	0.39	0.029	0.93
Age of the farmer	0.156	0.63	–0.119	0.71	–0.267	0.40
Length of animal farming experience of the farmer	0.088	0.79	–0.168	0.60	–0.339	0.28
Level of education of the farmer	–0.404	0.19	–0.625	0.030	0.047	0.88

Figure S1. Scatter-plot of incidence of isolation of multidrug-resistant staphylococci from cases of subclinical mastitis during this longitudinal study throughout a milking period in 12 sheep flocks in Greece and age of newborns when taken away from the dam (size of circles corresponds to the number of antibiotics used for the treatment of mastitis in the farm).

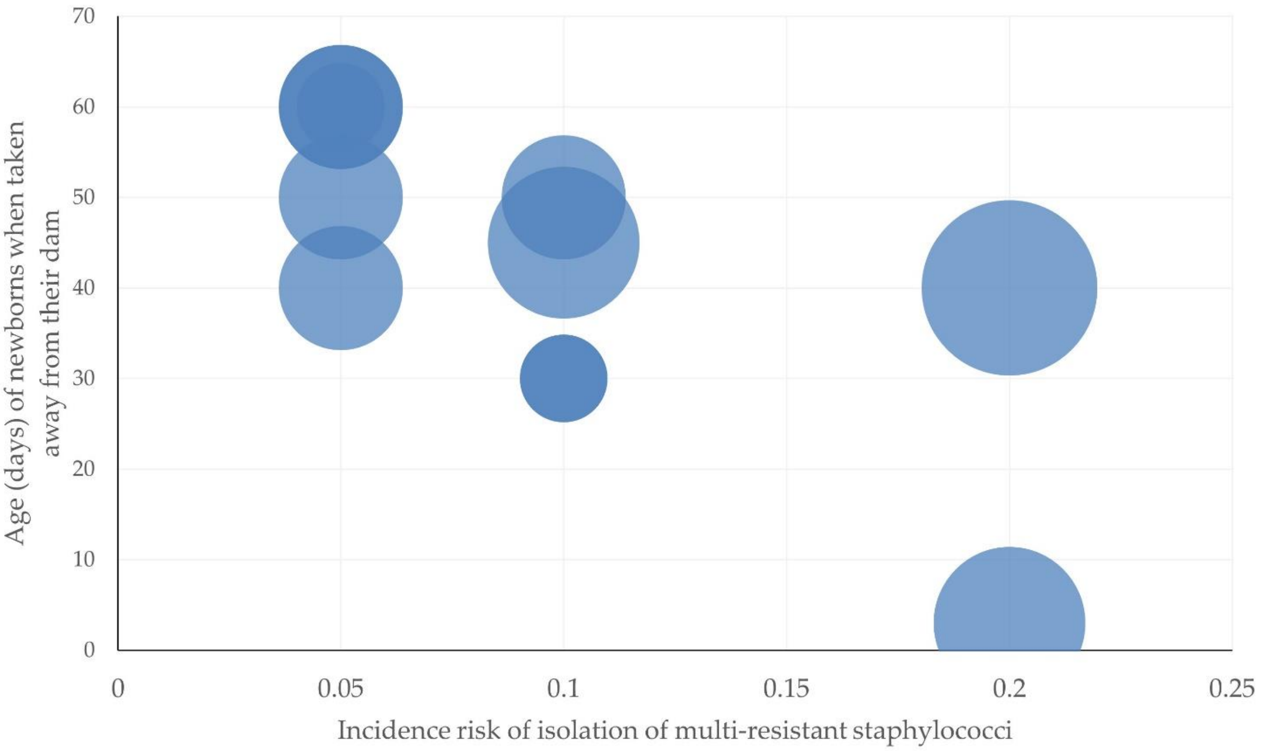

Table S5. Results of biofilm formation by staphylococcal isolates from cases of subclinical mastitis among 12 sheep flocks in Greece monitored throughout a milking period.

Bacterial identity	No. of bacterial isolates	No. (proportion) of biofilm-forming isolates
S. aureus	38	32 (84.2%)
S. capitis	2	1 (50.0%)
S. caprae	6	3 (50.0%)
S. chromogenes	24	18 (75.0%)
S. epidermidis	28	11 (39.3%)
S. equorum	4	2 (50.0%)
S. haemolyticus	4	2 (50.0%)
S. hominis	5	4 (80.0%)
S. lentus	9	3 (33.3%)
S. saprophyticus	1	1 (100.0%)
S. schleiferi	1	0 (0.0%)
S. sciuri	3	1 (33.3%)
S. simulans	34	26 (76.5%)
S. warneri	2	1 (50.0%)
S. xylosus	18	12 (66.7%)
Total	179	117 (65.4%)

Table S6. Contingency table indicating associations between anti-staphylococcal mastitis vaccination status and biofilm formation, according to antibiotic resistance, of staphylococcal isolates recovered from subclinical mastitis among 12 sheep flocks in Greece monitored throughout a milking period.

(a) Antibiotic-resistant isolates (*n* = 57)

		Anti-staphylococcal mastitis vaccination	
		+	-
Biofilm formation by staphylococcal isolates	+	15 (26.3%)	19 (33.3%)
	-	20 (35.1%)	3 (5.3%)

(b) Non antibiotic-resistant isolates (*n* = 122)

		Anti-staphylococcal mastitis vaccination	
		+	-
Biofilm formation by staphylococcal isolates	+	49 (40.2%)	34 (27.9%)
	-	32 (26.2%)	7 (5.7%)

Table S7. Details of 12 flocks included in this longitudinal study of subclinical mastitis in Greece.

Farm	Location	Management system	No. of ewes	Breed of ewes	Start of lambing period	Annual milk production per ewe	Machine-milking	Milking sessions daily	Sampling period
1	Achaea, Western Greece	Semi-extensive	130	Friesian	September	154 L	Yes	Two	4 visits: November 2019 to June 2020
2	Achaea, Western Greece	Semi-extensive	170	Friesian	October	235 L	Yes	Two	4 visits: December 2019 to June 2020
3	Achaea, Western Greece	Semi-extensive	180	Local	September	144 L	Yes	Two	4 visits: November 2019 to June 2020
4	Achaea, Western Greece	Intensive	235	Local	October	111 L	Yes	Two	4 visits: December 2019 to June 2020
5	Achaea, Western Greece	Semi-intensive	150	Lacaune	September	167 L	Yes	Two	4 visits: November 2019 to June 2020
6	Achaea, Western Greece	Semi-intensive	230	Chios	September	296 L	Yes	Two	4 visits: November 2019 to June 2020
7	Corinthia, Peloponnese	Semi-intensive	300	Local	September	293 L	Yes	Three	4 visits: November 2019 to June 2020
8	Corinthia, Peloponnese	Semi-intensive	85	Assaf	October	400 L	Yes	Two	4 visits: December 2019 to June 2020
9	Corinthia, Peloponnese	Intensive	600	Chios	October	183 L	Yes	Three	4 visits: December 2019 to June 2020
10	Corinthia, Peloponnese	Semi-intensive	230	Assaf	September	357 L	Yes	Three	4 visits: November 2019 to June 2020
11	Corinthia, Peloponnese	Semi-intensive	280	Lacaune	September	232 L	Yes	Two	4 visits: November 2019 to June 2020

12	Corinthia, Peloponnese	Semi-intensive	145	Local	October	152 L	Yes	Two	4 visits: November 2019 to June 2020

Table S8. Concentrations of antibiotics against which resistance of staphylococcal isolates was tested in the automated system BD Phoenix™ M50.

Antibiotics	Antibiotic concentrations (mg L^{-1})
amikacin	4, 8, 16
ampicillin	2, 4, 8
ciprofloxacin	0.25, 0.5, 1, 2, 4
clindamycin	0.25, 0.5, 1
erythromycin	0.25, 0.5, 1, 2
fosfomycin	16, 32, 64
fucidic acid	1, 2, 4, 8
gentamicin	1, 2, 4
moxifloxacin	0.25, 0.5, 1, 2
mupirocin	1, 2, 4
oxaxillin	0.25, 0.5, 1, 2
penicillin G	0.0625, 0.125, 0.25
rifampicin	0.25, 0.5, 1
teicoplanin	1, 2, 4
tetracycline	0.5, 1, 2
tobramycin	1, 2, 4
trimethoprim-sulfamethoxazole	1, 2, 4
vancomycin	0.5, 1, 2

Table S9. Detailed description of the criteria for definition of subclinical mastitis in sheep flocks.

Subclinical mastitis was defined in ewes, in which:
(1) a bacteriologically positive mammary secretion sample: [a] > 10 colonies of the same organism and [b] no more than two different types of colonies,
(2) with concurrently increased cell content: [a] CMT score ≥ '1' and [b] neutrophil and lymphocyte proportion cumulatively ≥ 65% of all leucocytes, was detected,
(3) with no presence of abnormal gross findings in the mammary gland (including changes in secretion).

Table S10. Variables (*n* = 20) evaluated for potential association with the isolation of antibiotic-resistant staphylococci from cases of subclinical mastitis during this longitudinal study throughout a milking period in 12 sheep flocks in Greece.

Management system applied in the flocks
No. of ewes in the flock
Average age of culling females
Month of the start of the lambing season
Total visits made annually by veterinarians to the flock during the preceding season
Use of laboratory diagnostic examinations in samples of milk preventively
Age of lamb removal from their dams
Daily number of milking sessions
Duration of the dry-period
Administration of selenium to pregnant ewes
Administration of 'dry-ewe' treatment at the end of the lactation period
Use of teat disinfection after milking
Number of antibiotics used for treatment of mastitis
Route for administration of antimicrobials
Vaccination against staphylococcal mastitis
Annual frequency of systemic disinfections in the farm
Temperature of cleaning water in the milking parlour
Age of the farmer
Length of animal farming experience of the farmer
Level of education of the farmer

Table S11. Details of multivariable models employed for the evaluation of risk factors for the isolation of antibiotic-resistant staphylococci from cases of subclinical mastitis during this longitudinal study throughout a milking period in 12 sheep flocks in Greece.

Outcome evaluated in each model of multivariable analysis	Number of variables offered to each multi-variable model	Variables used in the final round of back-wards elimination during each model of multivariable analysis
Isolation of staphylococci from cases of subclinical mastitis, resistant to at least one (any) antibiotic	4	(a) Vaccination against staphylococcal mastitis, (b) Level of education of the farmer
Isolation of oxacillin-resistant staphylococci from cases of subclinical mastitis	2	(a) Administration of selenium to pregnant ewes, (b) Vaccination against staphylococcal mastitis
Isolation of multidrug-resistant staphylococci from cases of subclinical mastitis	5	(a) Month of the start of the lambing season, (b) Age of lamb removal from their dams, (c) Number of antibiotics used for treatment of mastitis
